# Experimental and Numerical Evaluation on Deformation and Fracture Mechanism of Cast Duplex Stainless Steel Tubular Specimen

**DOI:** 10.3390/ma13153430

**Published:** 2020-08-04

**Authors:** Zhenhua Li, Xinyu Wang, Tao Chen, Fan Feng, Pan Liu, Yonghao Lu

**Affiliations:** 1 State Key Laboratory of Nuclear Power Safety Monitoring Technology and Equipment, China Nuclear Power Engineering Co., Ltd., Shenzhen 518172, China; chent2@cgnpc.com.cn (T.C.); liup@cgnpc.com.cn (P.L.); 2 National Center for Materials Service Safety, University of Science and Technology Beijing, Beijing 100083, China; zhenhuali_ustb@163.com (Z.L.); wangxinyu-010@163.com (X.W.); fengfan0714@163.com (F.F.)

**Keywords:** tubular specimen, fracture mechanism, numerical evaluation, cast duplex stainless steel

## Abstract

The deformation behavior and fracture mechanism of cast duplex stainless steel tubular specimens under different tensile stages were investigated through experimental and numerical evaluation. The results showed that the axial stress was redistributed due to the necking of the tubular specimen, the axial stress near the internal wall was larger than those near the external wall, and its maximum axial stress was distributed between the internal wall and the center of the wall thickness. Microcracks and voids were initiated under the maximum shear stress along the δ/γ phase interface and propagated to the ferrite interior. The voids were connected and merged into the main crack through the propagation of the microcracks. Moreover, the main crack first propagated to the internal wall and then rapidly propagated to the external wall. The fracture morphology can be divided into three types: shear lip zones that can be found on both the internal and external walls, and shear lip zones that can be found on either only the internal wall or the external wall.

## 1. Introduction

Cast duplex stainless steels (CDSSs) have excellent mechanical properties, corrosion resistance, and good weldability, which are widely used in the primary circuit piping of pressurized water reactors (PWRs) [1,2,3,4]. It is particularly important for the safety operation of nuclear power plants to ensure the integrity of the primary circuit pressure boundary. The deformation behavior and fracture mechanism of CDSSs are of great significance for its service and improvement of the material production process, and the tensile test is the most basic test for studying the mechanical properties of materials. During the last years, some authors have studied the deformation mode and microstructure evolution of CDSSs under tensile loading by means of various methods [5,6,7,8,9]. Guo et al. considered that the slip lines first appear in the austenite matrix, and slip lines in ferrite can be caused by deformation in austenite or the bulk deformation of the ferrite itself [5]. Voids tended to form at the ferrite–austenite interphase boundaries or around the inclusions [5,6]. Dislocation in austenite piled up against the phase boundary and created a local stress concentration, which generated dislocation in ferrite [7]. At present, many studies have been conducted on the microstructural evolution and mechanical behavior of CDSSs during long-term thermal aging [10,11,12]. The main reason is that the ferrite phase in CDSSs will decompose into a Fe-rich α phase and Cr-rich α’ phase, which will cause ferrite phase embrittlement. However, the effect of the low-fluence neutron irradiation on variation is neither significant nor apparent for the thermally aged steels [2]. Moreover, the fatigue behavior of various duplex stainless steels was studied [13,14,15,16]. The role of grain and phase boundaries has been shown to be critical not only in the fatigue crack initiation but also in the crack propagation stage.

At present, the research on the tensile test of CDSSs is mainly focused on the bar or plate specimens, while the actual service CDSSs are tubular specimens with a special hollow structure. The deformation behavior and fracture mechanism may be different from that of the bar or plate specimens, but there are few related studies [17]. In this study, a kind of Z3CN20-09M tubular tensile specimen was designed to study the microstructural evolution and fracture mode of CDSSs under different deformation variables. Moreover, the damage model of the tubular specimen was established, and the numerical analysis was carried out. The deformation behavior and fracture mechanism are compared and evaluated by a tensile experiment and numerical simulation. It provides a reliable theoretical basis for the production process and service evaluation of such tubular specimens.

## 2. Experimental and Simulation

### 2.1. Test Material

**Figure 1:**
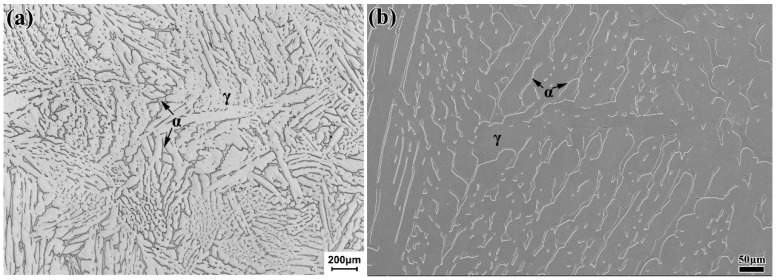
Microstructure of Z3CN20-09M stainless steel under (**a**) optical microscope (OM) and (**b**) scanning electron microscope (SEM).

The material investigated in this study was Z3CN20-09M, which was obtained from the primary circuit piping of PWR. The process of solution treatment was water quenching after holding at 1180 °C for 8 h. The chemical composition of the material is shown in Table 1. The original structure of the material was island ferrite with different shapes distributed on the austenite matrix, as shown in Figure 1. The volume fraction of ferrite in this material was calculated by metallographic statistics method to be 17.57%.

### 2.2. Test Conditions and Methods

**Figure 2:**
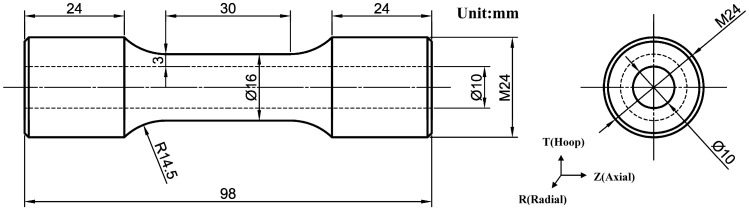
Schematic diagram of designed tensile tubular specimen.

The uniaxial tensile test adopts ø 16 mm × 3 mm hollow tubular specimen, and its shape and detailed dimensions are shown in Figure 2. The specimens were processed from the center of 1/4 wall thickness of primary circuit piping, and its axial direction was parallel to that of piping. In order to ensure the reliability of the tensile test, the last internal wall machining process of the tubular specimen adopted the reaming process to provide the surface roughness of the internal wall and remove the working hardening layer of the internal wall. All the tensile tests were carried out at room temperature in air on an MTS-810 electro-hydraulic servo fatigue testing machine (MTS Systems Corporation, Eden Prairie, MN, USA), and the tensile strain rate was 2.5 × 10^−4^ s^−1^. In order to study the deformation behavior and fracture mechanism of the tubular specimens under different tensile deformations, three specimens and tensile test parameters of the same specifications were used in each group, and the tests were interrupted when stretching to different stages. Table 2 shows the elongation of the gauge section when the test was stopped.

### 2.3. Microstructural Characterization

After being grounded and polished with water abrasive paper, the metallographic specimens were slightly electrolytic eroded with 10% oxalic acid to clearly show the interface between austenite and ferrite. The original structure of Z3CN20-09M and microstructure characteristics under different tensile deformations were observed by OM (Olympus Corporation, Tokyo, Japan). The ImageJ software (v.1.8.0, National Institutes of Health, Bethesda, Rockville, MD, USA) was used to quantitatively measure the ferrite content in the OM image. After the tensile test, SEM (Carl Zeiss, Oberkochen, Germany) was used to observe the surface fracture morphology of the tensile specimens. In addition, transmission electron microscope (TEM, Tecnai G2 F20, FEI, Portland, OR, USA) analysis was carried out on the tubular specimens with different tensile stages from the uniform deformation part. The thickness of the specimens was reduced by the electrolytic double spray method. The operating voltage was 25 V, and the solution was a mixture of 10% perchloric acid and 90% methanol.

### 2.4. Simulation Methods

**Figure 3:**
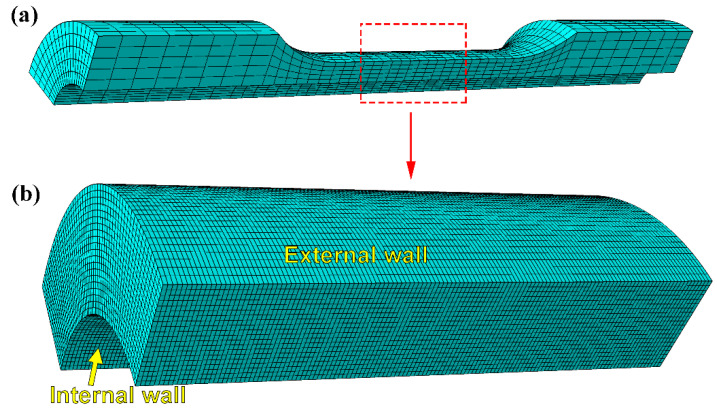
Finite element mesh of 1/4 tubular specimen (**a**) and the sub model (**b**).

The finite element analysis (FEA) software ABAQUS (v.2016, Dassault Systèmes, Vélizy-Villacoublay, France) was used to simulate the tensile deformation and fracture behavior of the tubular specimen with the size of Figure 2. In order to simplify the calculation, the quarter tubular specimen was modeled as shown in Figure 3a. A section of arc with a length of 20 mm in the necking part of the gauge position was defined as a sub model for analysis (Figure 3b), so as to achieve high calculation accuracy. The FEA model consisted of 134,000 linear hexahedron elements of type C3D8R. The material model parameters in the FEA process are shown in Table 3. The constitutive relation or hardening rule is considered as the Johnson–Cook (J-C) model, which means that the equivalent stress *σ_eq_* as a function of plastic strain, strain rate, and temperature given by the relation.

(1) $$\sigma_{eq}=(A+B\varepsilon_{eq}{}^{n})\cdot \left(1+C\ln{\dot{\varepsilon }}_{eq}^{*}\right)\cdot \left(1-T^{*}{}^{m}\right).$$

where *A*, *B*, *n*, *C,* and *m* are the model constants, *ε_eq_* is the equivalent plastic strain, ${\dot{\varepsilon }}_{eq}^{*}$ = ${\dot{\varepsilon }}_{eq}$/${\dot{\varepsilon }}_{0}$ is the dimensionless equivalent plastic strain rate, and $T^{*}=\left(T-T_{r}\right)/\left(T_{m}-T_{r}\right)$ is the dimensionless temperature.

The ductile criterion is a phenomenological model for predicting the onset of damage due to the nucleation, growth, and coalescence of voids. The model assumes that damage accumulates in the material element during plastic straining, which accelerates immediately when the damage reaches a critical value [18].

(2) $$D=\int_{0}^{{\bar{\varepsilon }}_{f}}\frac{1}{f\left(\sigma^{*}\right)}d\bar{\varepsilon }$$

where *D* is defined as a damage variable that varies between 0 (material not damaged) and 1 (fully failed material), ${\bar{\varepsilon }}_{f}$ is the equivalent fracture strain, $\sigma^{*}=\sigma_{m}/\sigma_{eq}$ is the stress triaxiality ratio, and *σ_m_* is the mean stress.

## 3. Results

### 3.1. Tensile Properties

**Figure 4:**
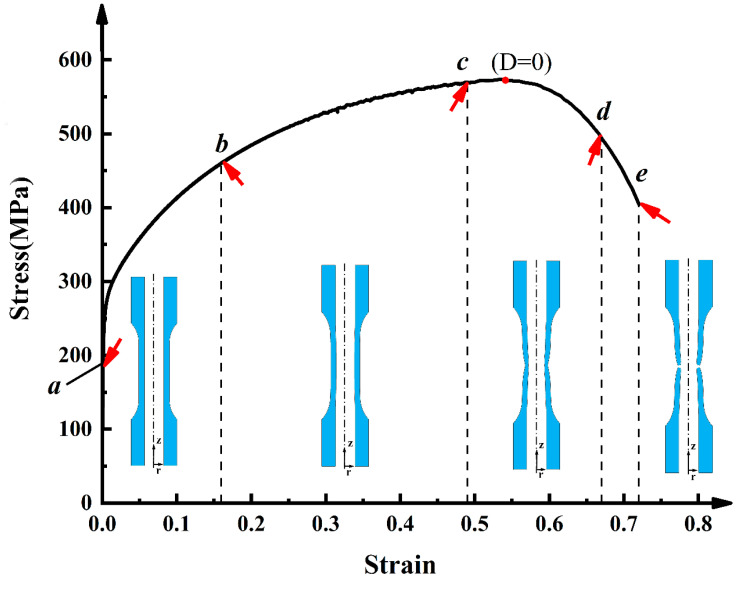
Engineering stress–strain curve of tubular specimen.

The complete tensile stress–strain curve of the tubular specimen is shown in Figure 4. It can be seen that there is no obvious yield plateau in the stress–strain curve of the material, and the stress value corresponding to the yield strength of 0.2% plastic strain is 254.5 MPa. In Figure 4, a–e respectively represent the position of the deformation amount in the complete stress–strain curve when the test specimens are interrupted. Among them, a is the original specimen without tension, b is the interrupted test specimen when stretching to 16%, and c is the interrupted test specimen when stretching to 49%. It can be seen that both b and c specimens are in the uniform deformation stage, and the tensile strength is 573.6 MPa. d is the interrupted test specimen when it is stretched to 67% and the specimen has an obvious necking phenomenon. e is the specimen stretched to complete fracture separation, and its elongation is 72%.

### 3.2. Fracture Morphology

**Figure 5:**
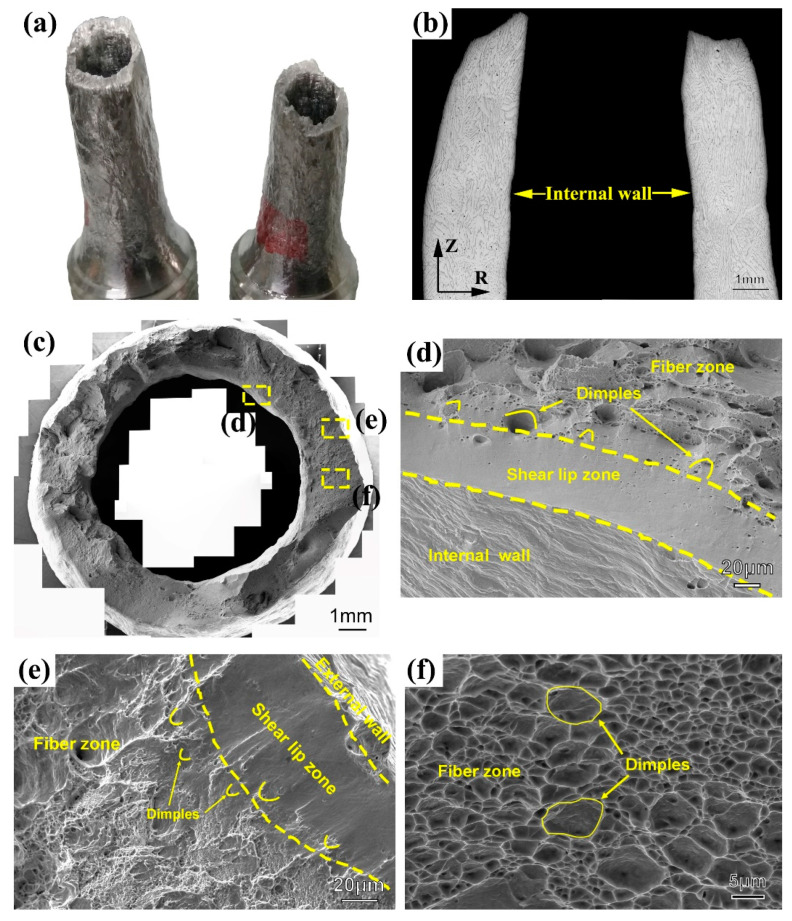
(**a**) Overall fracture morphology of the tubular specimen. (**b**) R-Z section of fracture microstructure, (**c**) fracture surface morphology, (**d**) shear lip zone around internal wall, (**e**) shear lip zone around external wall, (**f**) fiber zone morphology of fracture surface.

Figure 5 shows the fracture morphology of the complete tensile fracture tubular specimen. The overall morphology of the tubular specimen is gray and shear fracture (Figure 5a). The R-Z section of the fracture microstructure is observed by OM (Figure 5b). The grains are elongated along the axial direction, and the direction of ferrite grains tended to be consistent with the axial direction. The fracture surface morphology in the R-T direction was observed by SEM. As shown in Figure 5c, it can be seen that the fracture is a typical ductile fracture that consists of a fiber zone and shear lip zone. The shear lip zone accounts for a small proportion, which is distributed discontinuously near the internal wall (Figure 5d) and external wall (Figure 5e). Most of the other zones are fiber zones composed of dimples (Figure 5f), and no radiation zone is found. In the flat fiber zone, it can be seen that the dimple opening has no obvious direction, while the dimple opening near the shear lip zone faces the shear lip. The above fracture morphology characteristics indicate that the fracture sequence is from the center of the wall thickness to the tube wall.

### 3.3. Crack Initiation and Propagation Behavior

**Figure 6:**
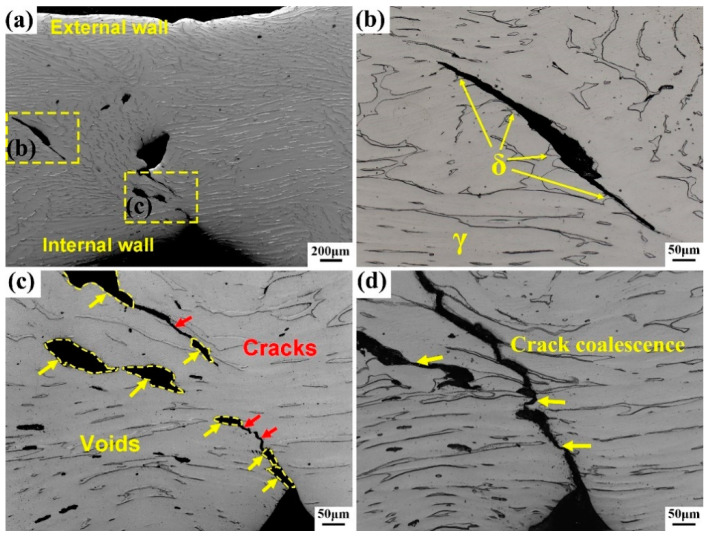
R-Z section of interrupted d specimen. (**a**) macroscopic deformation morphology, (**b**,**c**) are the magnification morphology of (**a**), (**d**) is the further polished image of (**c**).

In order to study the crack initiation and propagation behavior of the tubular specimen, the interrupted specimen d is observed by OM. Figure 6a shows the macroscopic deformation morphology of the R-Z section, whose surface shows orange peel-like wrinkles. This is due to the ferrite phase having different distribution forms in the austenite phase, such as strips, islands, etc., and the different forms of ferrite phase have different resistance to deformation, which plays a strengthening role on the austenite matrix, resulting in orange peel-like wrinkles on the original smooth specimen surface after deformation under loading. Figure 6b is a partial magnification morphology of Figure 6a. It can be seen from Figure 6b that the cracks’ initiation and propagation occur at the phase interface between ferrite and austenite, and propagation first occurs along the interior of ferrite. This is because austenite and ferrite have different slip directions and deformation amounts on both sides of the phase boundary. Most of the slip lines are blocked by the phase boundary after extending here and gather at the phase boundary and change the direction. The crack nucleates at the phase boundary when the amount of deformation reaches a certain degree. It propagates toward the ferrite phase, and cleavage fracture occurs in ferrite because of the poor toughness of the ferrite phase. The direction of crack propagation is 45° to the direction of tension stress, which is the direction of maximum shear stress. Figure 6c is a magnification morphology of the internal wall in Figure 6a. Cracks and voids of different sizes are distributed on the deformation surface, and they are mainly distributed on the inner side of the tube wall. The area of Figure 6c is further lightly polished again and then observed until Figure 6d. It can be seen that microcracks and voids grow further, which are connected and merged into main crack through the propagation of microcracks.

### 3.4. Deformation Microstructure Evolution

**Figure 7:**
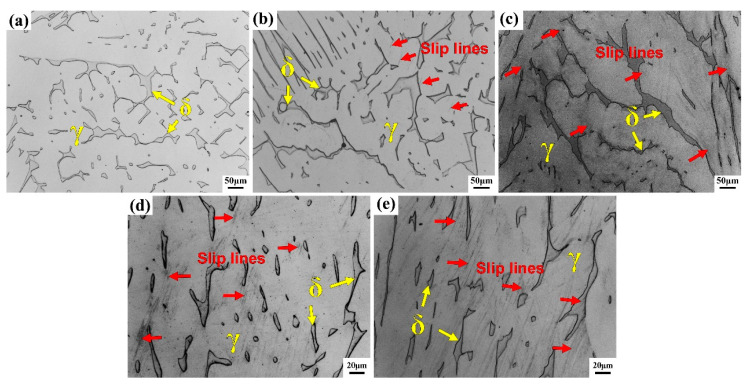
Microstructure of R-Z section at different tensile stages. (**a**) A = 0%, (**b**) A = 16%, (**c**) A = 49%, (**d**) A = 67%, and (**e**) A = 72%. (**d**,**e**) observed at non-necked part.

The microstructure of the interrupted specimens with different tensile deformation is observed in the uniform deformation areas, as shown in Figure 7. With the increase of deformation, the slip lines become more and more dense, and the slip direction develops from a single system slip to a multi-system slip, which are all distributed in the austenite grains (Figure 7b–d). Slip lines can also be found inside the ferrite, and the slip direction is single when the amount of deformation is large (Figure 7e). Austenite is a face-centered cubic (FCC) structure, the close packed surface is {111}, and the direction of the close packed surface is <110>. Single slip and cross-slip can occur when dislocation moves in the slip system under shear stress. However, ferrite is a body-centered cubic (BCC) structure, and the close packed direction is <111>. Any surface containing this close packed direction may be a potential slip surface. The specific slip surface is mainly determined by the crystal orientation relative to the deformation axis. The deformation of austenite around ferrite with a larger shape or network distribution is smaller; the deformation of austenite around ferrite with a small shape, thin strip, or island shape is larger. The direction of the slip lines in austenite are affected by the crystal orientation of the ferrite, which is parallel to the strip ferrite or at a minimum angle with the ferrite distribution direction.

**Figure 8:**
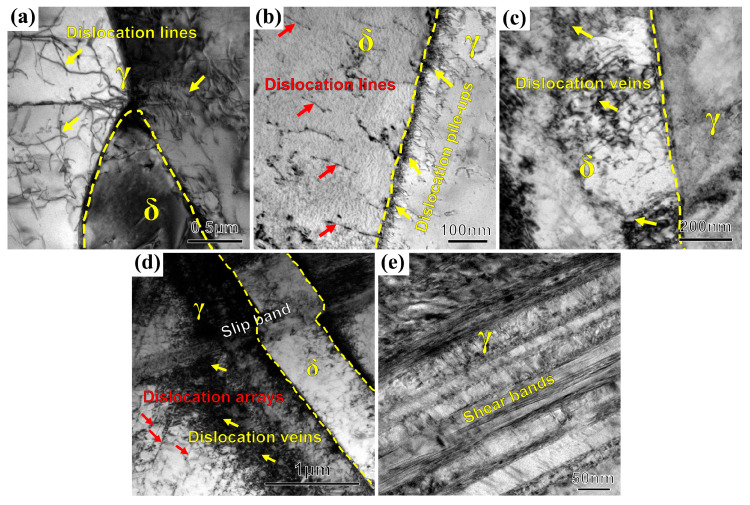
Evolution of dislocation morphology of R-Z section at different tensile stages. (**a**) A = 0%, (**b**) A = 16%, (**c**) A = 49%, (**d**) A = 67%, and (**e**) A = 72%.

TEM observation is carried out on the uniform deformation part of the tubular specimens with different tensile deformation, as shown in Figure 8. In the original specimen without tension, a relatively sparse distribution of dislocation lines can be observed in austenite and ferrite, in which the density of dislocation lines in austenite is significantly higher than that of ferrite (Figure 8a). With the increase of tensile deformation, the density of dislocation lines increases obviously, and it mainly grows along the phase boundary toward the interior of austenite (Figure 8b). Moreover, it can be found that stacking faults and dislocation lines are entangled together, forming a large number of dislocation pile-ups on the side of austenite. Due to the local stress concentration caused by dislocation pile-ups in austenite, dislocation sources are activated in the ferrite at the grain boundary. As shown in Figure 8c, the dislocation density is further increased, and dislocation veins with higher density are formed in ferrite. A large number of twin structures appear in the austenite grains, whose density increases with the increase of tensile deformation. Afterwards, dislocation veins gradually develop into dislocation arrays (Figure 8d). Many parallel and strongly deformed striated shear bands are formed on the interface (Figure 8e).

### 3.5. Finite Element Simulation

**Figure 9:**
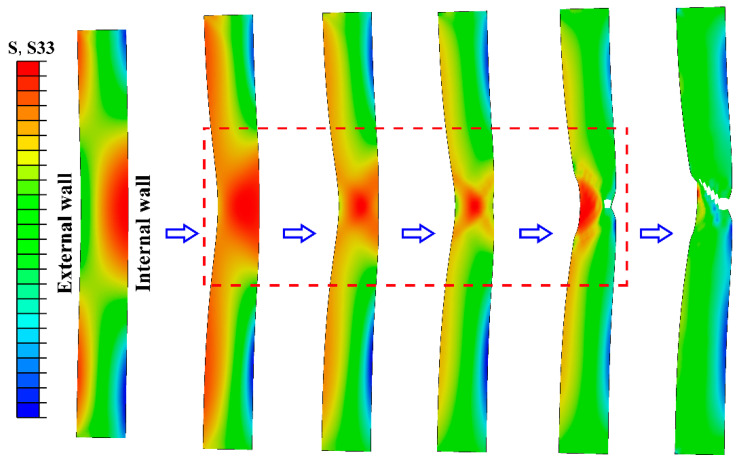
Axial stress (S33) contours during tensile necking of tubular specimen.

**Figure 10:**
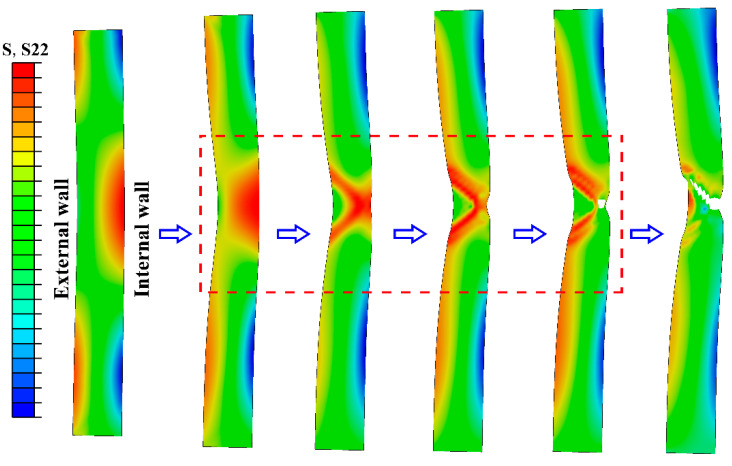
Hoop stress (S22) contours during tensile necking of tubular specimen.

The evolution of the axial stress contours in the tensile process of the tubular specimen is shown in Figure 9. In the uniform tensile stage, the tubular specimen is uniformly contracted inwardly while being extended. After necking, the axial stress near the internal wall of the tube has an obvious increasing trend. The axial stress at the center thickness of the tube wall is slightly smaller than the internal wall at the beginning and increases rapidly at the end of necking, while the axial stress near the internal wall rapidly decreases—that is, the peak position of the axial stress shifted from the internal wall to the center of the wall thickness. The axial stress of the external wall undergoes a fluctuation process which first decreases and then increases during the necking stage. Figure 10 shows the evolution of hoop stress contours during the tensile process of the tubular specimen. The hoop stress has a similar evolution process with the axial stress, but its peak position is always near the internal wall. It can be seen that the location of crack initiation is mainly concentrated between the internal wall and the center of wall thickness when the tubular specimen is necking, and the main crack first propagates to the internal wall and rapidly to the external wall.

## 4. Discussion

### 4.1. Deformation Behavior and Fracture Mechanism

According to the results of the test and finite element simulation, the original internal radius *r_i_* of the tubular specimen changed little in the uniform tensile stage. If the tensile loading at a certain time was *F_z_* and the external radius of the tubular specimen was *r*, the instantaneous axial stress of uniform tensile can be expressed as

(3) $$\sigma_{z}=\frac{F_{z}}{\pi r^{2}\left[1-{\left(r_{i}/r\right)}^{2}\right]}.$$

It was assumed that the gauge length of the original and at a certain time was *l*_0_ and *l*, and the strains *ε_z_*, *ε_θ_*, and *ε_r_* in the axial, hoop, and radial directions of tension can be expressed as

(4) $$\varepsilon_{z}=\ln\frac{l}{l_{0}}$$

(5) $$\varepsilon_{\theta }=\ln\frac{r-t/2}{r_{0}-t_{0}/2}$$

(6) $$\varepsilon_{r}=\ln\frac{t}{t_{0}}$$

where *t*_0_ was the original tube wall thickness, *t* was the instantaneous tube wall thickness, and *r*_0_ was the original tube external diameter.

According to the principle of constant volume *ε_z_* + *ε_θ_* + *ε_r_* = 0, the uniform tensile stage was a uniaxial stress state of axisymmetric deformation *ε_θ_* = *ε_r_*. It was easy to obtain the relationship between instantaneous wall thickness *t* and the tube external diameter *d*:

(7) $$t=\frac{t_{0}}{d_{0}}d$$

where *d*_0_ was the original external diameter of the tube. From *t*/*t*_0_ = *d*/*d*_0_, it can be seen that the thinning ratio of the tube wall was equal to the change ratio of the external diameter of the tube during the process of uniform tensile. In order to avoid measuring the wall thickness *t* when calculating the instantaneous stress, Formula (3) can be rewritten as

(8) $$\sigma_{z}=\frac{F_{z}}{\pi d^{2}\left(t_{0}/d_{0}\right)\left(1-t_{0}/d_{0}\right)}.$$

By testing the tensile loading *F_z_* and the external diameter *d* of the tube, the instantaneous real axial stress can be calculated directly by Formula (8). When the relative wall thickness of the tube was *t*_0_/*d*_0_ = 0.5, it will become the real stress in the tensile direction of the solid bar corresponding to the external diameter *d*.

(9) $$\sigma_{z}^{\prime }=\frac{4F_{z}}{\pi d^{2}}$$

Equation (9) was the true stress of the bar specimen in the uniform tensile stage. Different from that of the bar specimen, the tubular specimen had two free surfaces: the intrados surface and extrados surface. In the uniform tensile stage, when the loading did not exceed the tensile limit of the material, the tube body will contract evenly to the inner side while extending. The shrinkage was related to the plasticity of the material. When the loading exceeded the tensile limit of the material, necking occurred in the specimen, which was *ε_θ_* ≠ *ε_r_* ≠ *−ε_z_*/2, and the uniaxial stress state began to fail. The plastic deformation produced hoop and radial stresses of the tubular specimen, which made the stress state of the tubular tensile specimen change from simple axial stress to complex triaxial stress state after necking. The true axial stress *σ_z_* in the necking region was distributed unevenly along the minimum section (*z* = 0), which near the internal wall was larger than those near the external wall. Moreover, the other two stresses on the inner side of the minimum section were larger than the corresponding outer side stress value. At the same radius *r*, the axial stress was always the largest and the radial stress was the smallest, which was *σ_z_ > σ_θ_ > σ_r_*. The axial stress played a decisive role in the necking process in which the maximum stress was distributed between the internal wall and the center of the wall thickness. When the shear stress was 45° with the axial direction, the shear stress was the largest; that was the direction of the slip. Microcracks and voids were initiated under the maximum shear stress, which preferentially distributed along the ferrite and austenite phase boundaries, and propagated to the ferrite interior. It was also indicated by the finite element calculation and experimental results.

With the development of the tension process, microcracks grow gradually, and the propagation path folded along the direction of 45° with the axial direction, which was preferentially formed at the minimum section position of the necking section. The microvoids, which deviated from this minimum section position, were relatively large in size, but they have not preferentially merged with cracks near the internal wall. This was because the tubular specimen continued to load after microcracks were generated perpendicular to the tensile direction. Since the shear stress was the largest on the two planes at which the crack propagation front was at 45°, the microcracks actually propagated along one of the 45° planes first. After the propagation to a certain extent, the microcracks propagated along another 45° plane, since it cannot deviate from the minimum section of necking, until it returned to the original direction when it deviated from the minimum cross-section again.

**Figure 11:**
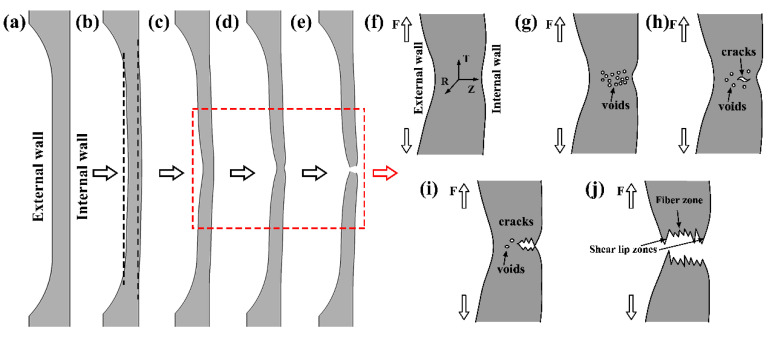
Deformation behavior and fracture mechanism of tubular specimen. (**a**) initial stage, (**b**) uniform tension, (**c**) necking, (**d**) severe necking, (**e**) fracture, (**f**–**j**) are the detailed deformation and fracture process of (**c**–**e**), in which (**f**) necking occurs, (**g**) microvoids appear, (**h**) microcracks are formed near the inner wall, (**i**) crack first propagates to the internal wall and (**j**) crack rapidly propagates to the external wall.

Under the action of triaxial stress, the voids were connected and merged into the main crack through the propagation of the microcrack. The main crack first propagated to the internal wall and then rapidly propagated to the external wall. Since the plastic deformation was irrecoverable and the strain changes of the internal and external walls were consistent, the crack rapidly propagated to the external wall direction. The deformation behavior and fracture mechanism are shown in Figure 11. When the cracks expanded to a certain extent, the heat generated by the plastic deformation leaded to the local softening of the metal. In addition, affected by the state of plane stress, it sheared off along the tensile axis in the direction of 45°, forming a cup-shaped fracture.

### 4.2. Fracture Morphology

**Figure 12:**
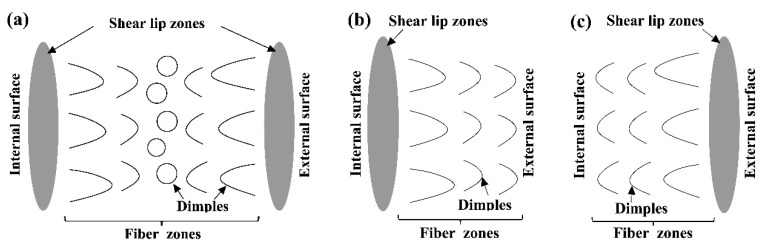
Classification of fracture morphology of tubular specimens. (**a**) Type A: Shear lip zones can be found on both the internal and external surface, (**b**) Type B: Shear lip zones can be found on the internal surface, (**c**) Type C: Shear lip zones can be found on the external surface.

According to the SEM results of fracture morphology (Figure 5), it can be found that the shear lips near the tube wall were not continuously distributed. This was because during the tubular specimen tensile test, affected by the test conditions and material characteristics, the crack propagation order of tube wall fracture at different positions in the circumferential direction of the tube was not consistent. The crack propagation sequence in the thickness direction of the tube wall will be affected by the inconsistency of the circumferential tangential tearing process, which made the fracture morphology of the tubular specimen have different characteristics. It can be divided into three types: shear lip zones can be found on both the internal and external walls of the tube wall direction, as shown in Figure 12a; shear lip zones can be found only on the internal wall (Figure 12b), or only near the external wall (Figure 12c).

## 5. Conclusions

The deformation behavior and fracture mechanism of Z3CN20-09M tubular specimens under uniaxial tension was investigated by experimental and numerical evaluation. The major results can be drawn as follows:

1. The crack initiated at the δ/γ phase interface, and first propagated in ferrite. The direction of the slip lines in austenite was affected by the crystal orientation of the ferrite, which was parallel to the strip ferrite or at a minimum angle with the ferrite distribution direction.

2. The location of crack initiation was mainly concentrated between the internal wall and the center of wall thickness when the tubular specimen was necking, and the main crack first propagated to the internal wall and rapidly to the external wall.

3. The fracture morphology of tubular specimens can be mainly divided into three types: shear lip zones that can be found both on the internal and external walls, and shear lip zones that can be found only on either the internal wall or the external wall.

## Figures and Tables

**Table 1:** Chemical composition of the Z3CN20-09M stainless steel (wt.%).

C	Si	Mn	P	S	Cr	Ni	Cu	Co	Mo	Fe
0.02	1.07	1.02	0.017	0.0023	20.16	8.93	0.063	0.026	0.22	Bal.

**Table 2:** Specimens at different tensile stages.

No.	Elongation (A)	Test Stage
a	0%	As-received
b	16%	Uniform deformation
c	49%	Before necking
d	67%	After necking
e	72%	After fracture

**Table 3:** Material model parameters in the finite element analysis (FEA) process.

*E*/GPa	*υ*	*A*/MPa	*B*/MPa	*n*	${\bar{\varepsilon }}_{f}$
190	0.3	254	820	0.657	0.66
